# Fluorescence *In Situ* Hybridization (FISH) Assays for Diagnosing Malaria in Endemic Areas

**DOI:** 10.1371/journal.pone.0136726

**Published:** 2015-09-02

**Authors:** Jyotsna Shah, Olivia Mark, Helena Weltman, Nicolas Barcelo, Wai Lo, Danuta Wronska, Srinivas Kakkilaya, Aravinda Rao, Shalia T. Bhat, Ruchi Sinha, Sabah Omar, Peter O’bare, Manuel Moro, Robert H. Gilman, Nick Harris

**Affiliations:** 1 ID-FISH Technology Inc., Palo Alto, California, United States of America; 2 IGeneX Inc. Palo Alto, California, United States of America,; 3 Nova Meditech Pvt. Ltd., Mangalore, India; 4 Kasturba Medical College Hospital, Mangalore, India; 5 Kenya Medical Research Institute, Nairobi, Kenya; 6 Walter Reed Project, Kisumu, Kenya; 7 National Institute of Health, Bethesda, Maryland, United States of America; 8 Johns Hopkins University, Baltimore, Maryland, United States of America; Royal Tropical Institute, NETHERLANDS

## Abstract

Malaria is a responsible for approximately 600 thousand deaths worldwide every year. Appropriate and timely treatment of malaria can prevent deaths but is dependent on accurate and rapid diagnosis of the infection. Currently, microscopic examination of the Giemsa stained blood smears is the method of choice for diagnosing malaria. Although it has limited sensitivity and specificity in field conditions, it still remains the gold standard for the diagnosis of malaria. Here, we report the development of a fluorescence in situ hybridization (FISH) based method for detecting malaria infection in blood smears and describe the use of an LED light source that makes the method suitable for use in resource-limited malaria endemic countries. The *Plasmodium* Genus (P-Genus) FISH assay has a *Plasmodium* genus specific probe that detects all five species of *Plasmodium* known to cause the disease in humans. The *P*. *falciparum* (PF) FISH assay and *P*. *vivax* (PV) FISH assay detect and differentiate between *P*. *falciparum* and *P*. *vivax* respectively from other *Plasmodium* species. The FISH assays are more sensitive than Giemsa. The sensitivities of P-Genus, PF and PV FISH assays were found to be 98.2%, 94.5% and 98.3%, respectively compared to 89.9%, 83.3% and 87.9% for the detection of *Plasmodium*, *P*. *falciparum* and *P*. *vivax* by Giemsa staining respectively.

## Introduction

Human malaria a serious, often fatal, parasitic disease is caused by four *Plasmodium* species, *P*. *falciparum*, *P*. *vivax*. *P*. *malariae and P*. *ovale* that are transmitted from human to human by mosquito vectors of the genus *Anopheles*. The non-human primate malaria parasites *P*. *knowlesi*, *P*. *cynomolgi*, *P*. *brasilianum* and *P*. *simium* can also naturally infect humans to cause serious disease, and their accurate identification at the species level commonly requires PCR-based methods [1]. According to the WHO, there were approximately 198 million cases of malaria and an estimated 584,000 deaths in the world in 2013 [2]. About 3.3 billion people are at risk of malaria transmission. Africa is the most affected continent with more than 90% of all malaria deaths. South East Asia and the Eastern Mediterranean Region represent 6% and 3% of all the malaria death cases, respectively [3].

Microscopic examination of Giemsa stained thick and thin blood smears is the most widespread technique used for malaria diagnosis [4]. An experienced microscopist can detect densities as low as 5–10 parasites per μl of blood by this method, but the detection capabilities of a typical microscopist might be more realistically 50–100 parasites per μl of blood [5, 6]. Although Giemsa stained smears can be used to assess the parasite load as well as to monitor antimalarial treatment, it has several limitations: (1) it takes about 15 minutes to read a Giemsa stained smear; (2) it is not clearly able to differentiate dying parasites from live parasites; (3) it normally has limited sensitivity and specificity; and (4) it requires a trained microscopist.

Malaria antigen-based rapid diagnostic tests (RDTs) detect parasite specific antigens or enzymes by immunochromatography and have some ability to differentiate among the species of *Plasmodium*. RDTs offer the advantage of quick diagnosis, but often give false positive or false negative results [7–10]. They do not eliminate the need for standard Giemsa tests [11], because RDTs cannot quantify malaria parasites [5]. In US, all RDT results have to be confirmed by microscopy. WHO recommends that anti-malarial treatment should only be limited to confirmed positive cases. However, treatment based on clinical suspicion should be considered when a parasitological diagnosis is not accessible within two hours of sample collection [3].

PCR is a very sensitive diagnostic method for detection and species identification of malaria parasites. It can detect as few as 1–5 parasites/μl of blood. Thus, it is useful for diagnosis of patients with low levels of parasitemia or mixed infections. It has gained acceptance to confirm malaria cases, monitor treatment and identify drug resistance in resource-rich settings [5]. Currently there are no FDA approved PCR tests and therefore in the US PCR is usually used for cases where blood film diagnosis is inconclusive or for identification of *Plasmodium* species in reference laboratories and health departments [12]. Giemsa stained smear microscopy when compared with PCR has a sensitivity between 50% [13] and 93% [14]. Despite being more accurate, PCR is time-consuming and expensive. Therefore it is generally not used in the initial diagnosis and treatment of patients with malaria [4].

Fluorescence in situ hybridization (FISH) is a cytogenetic technique used to detect and localize the specific nucleic acid (DNA or RNA) sequences by hybridizing with complementary sequences labeled with fluorescent probes. In 1989, Delong *et al*. demonstrated that FISH can be used to detect a single microbial cell using fluorescent labeled oligodeoxynucleotides complementary to 16S ribosomal RNA (rRNA), and that with appropriate probes labeled with different fluorescent dyes, FISH can distinguish closely related organisms [15]. In the last 25 years there have been several publications describing FISH technology for the detection and differentiation of infectious agents in cultures and clinical samples [15–26]. Shah *et al*. (1995) demonstrated that FISH can detect *Pneumocystis carinii* in patient’s induced sputum sample and touch-prep tissues, and that multiple probes labeled with different fluorescent dyes can distinguish different strains of *P*. *carinii* in tissues [17]. Subsequently a Babesia FISH assay for direct detection of *Babesia* in a thin blood smear using a fluorescent labeled oligomer probe, targeted to *B*. *microti* 18S rRNA was developed [18]. Several FISH assays using peptide nucleic acid (PNA) probes and DNA probes have been described for culture confirmation of pathogens and for direct detection of *M*. *tuberculosis* and *M*. *avium* complex in clinical samples [19–25].

Although in resource-rich countries, FISH using PNA probes has been used for culture confirmation for some pathogens for several years, *Babesia* FISH is currently the only FISH test used for clinical diagnosis. Requirement of a fluorescence microscope with appropriate filters to read processed FISH smears may have hindered the development of FISH assays for diagnosis of infectious diseases such as malaria, in resource-poor countries. Mercury lamp fluorescence microscope (powered by white light sources that generate many intense bands for fluorescence excitation across the UV-visible light spectrum) commonly used for FISH is expensive to buy and maintain for the following reasons. Mercury bulbs are very expensive. Mercury lamp intensity deteriorates over time. At around 100+ hours, the microscope images often begin to skew. At 200+ hours the bulb has to be replaced. Every time the bulb is changed, bulb alignment is required, since mercury lamp has an uneven illumination across the microscope field of view. In addition, mercury is a health hazard; therefore, the mercury bulbs have to be disposed as hazardous waste.

Light-emitting diode [LED] is a versatile semiconductor device that possess many advantages over mercury arc lamps. LEDs are efficient enough to be powered by low voltage batteries or relatively inexpensive switchable power supplies. They have a diverse spectral output, and thus it is possible to select an individual diode light source to supply the optimum excitation wavelength band for fluorophores spanning the ultraviolet, visible, and near-infrared regions. LEDs have the following advantages over mercury lamps: (1) they offer a very distinct excitation peak, (2) they can be individually switched on and off without delay, and do not require a warm-up and cool down time as in a mercury arc lamp, (3) they have a lifetime of 10,000+ hours with no decay curve, (4) since there is no mercury bulb involved, focus adjustments is not necessary and there are no health hazard issues or maintenance cost, (6) The newer high-power LEDs generate sufficient intensity to provide a useful illumination source for a wide spectrum of applications in fluorescence microscopy. Therefore we have evaluated the performance of a LED light source with appropriate filters attached to a regular light microscope to read processed FISH smears for potential use in resource-poor malaria-endemic countries.

Here we report the development of a simple and specific FISH assay that can be used to diagnose and monitor treatment responses in resource-poor malaria endemic countries and the evaluation of a LED light source with a blue-green filter set that can be attached to a standard light microscope with 100X objective, to read FISH processed smears.

Ribosomal RNA (rRNA) was used as a target for developing FISH assays for diagnosing malaria for the following reasons: (1) rRNA is highly abundant in the cell cytoplasm and therefore can be visualized using sequence-specific fluorescence probes under a microscope, without target sequence amplification, and (2) As a consequence of variable nucleotide sequence conservation, it is possible to find short nucleotide stretches that are unique to the genus, species, sub-species or strain [27].

## Materials and Methods

### FISH Assays

*Plasmodium* genus FISH (P-Genus FISH) kit, (catalogue# PlasGK04), *P*. *falciparum* FISH (PF-FISH) kit, (catalogue #PfalK04), and *P*. *vivax* FISH (PV-FISH) kit, (catalogue# PvivK04), used for the study were provided by ID-FISH Technology Inc., Palo Alto, CA, USA. All assay kits contained relevant rRNA-specific probes, Smear Preparation Reagent (SPR), 2.5x Plasmodium Wash Buffer, 10x Plasmodium Rinse Buffer and Plasmodium Counterstain. The assays were performed according to the manufacturer’s instructions provided with the kits.

#### Human blood samples

Only previously collected left-over de-identified blood samples (that would otherwise be discarded) of patients suspected of malaria or had malaria-like symptoms, were used for the study. Samples were collected at four sites, IGeneX Inc., Palo Alto, California, USA (IGeneX), Walter Reed-KEMRI, Kisumu, Kenya (Walter Reed), Kasturba Medical College Hospital, Mangalore, India (KMC) and hospitals in Iquitos, Peru (Peru). The study was reviewed and approved by the ethics committee of Walter Reed, KMC and Universidad Peruana Cayetano Heredia, and Asociación Benéfica PRISMA, both in Lima, Peru; by the Directorate of Health, Iquitos, Peru; and by the Institutional Review Board of Johns Hopkins Bloomberg School of Public Health Institutional Review Board. In US ethical review board approval is not necessary for use of de-identified human samples that would otherwise be discarded. Therefore for the use of left-over de-identified blood samples from IGeneX, no ethical review approval was required. Since only left over de-identified blood samples or unstained methanol fixed thin smears prepared from capillary blood without anticoagulant were used, patient consent was not necessary. Each site prepared the smears from EDTA whole blood as described under smear preparation. In addition, each site provided Giemsa and RDT results. Bionow (Alere Medical, Gurgaon, India) *P*. *vivax* and *P*. *falciparum* RDT tests were used in India and IGeneX, while Paracheck RDT (Orchid Biomedical Systems, Goa, India) tests were used in Kenya and Peru.

Clinical study was performed on methanol-fixed smears prepared from venous blood of 357 patients (ranging in age from 1 year to 65 years), living in malaria endemic areas, as well as 150 samples from individuals with malaria-like symptoms from the United States (US), a country not endemic for malaria. Giemsa and RDT results were provided by the collection sites. All methanol fixed smears from patients were tested blindly by P-Genus, PF and PV FISH assays. Methanol fixed blood smears prepared from left over de-identified EDTA whole blood samples from 302 patients from Mangalore, India and surrounding areas were tested at KMC. 28 samples from Western Kenya, 27 samples from Iquitos, Peru and 150 samples from US were tested in our laboratory. This included 152 (50 *P*. *falciparum*; 101 *P*. *vivax* and one mixed infection) Giemsa positive samples ranging <100 to >10,000 parasites/μl blood (<100 13.8%; >100–35.5%; >1000–32.9%; and >10,000–17.8%).

#### FISH Assay Procedure

A set of four smears was prepared from EDTA whole blood. EDTA whole blood was mixed with SPR, 3 parts blood: 1 part SPR by volume. Each thin smear was prepared from 4 μl of the mixture, air-dried and fixed with methanol. Each sample was tested by P-Genus, PF and PV FISH assays. Briefly, after addition of 12 μl of appropriate hybridization buffer with probe mix to each methanol-fixed smear, the smear was covered with a plastic cover-slip and placed in a 37°C humid chamber for 30 minutes for hybridization. After 30 minutes, each smear was washed twice for 2 minutes each with 1x Wash Buffer at room temperature, followed by a rinse with 1x Rinse Buffer. After drying the smears in complete darkness, a drop of counterstain was added to each smear. Each smear was then covered with a glass cover-slip and viewed in a fluorescence microscope at 1000X magnification.

**Fig 1:**
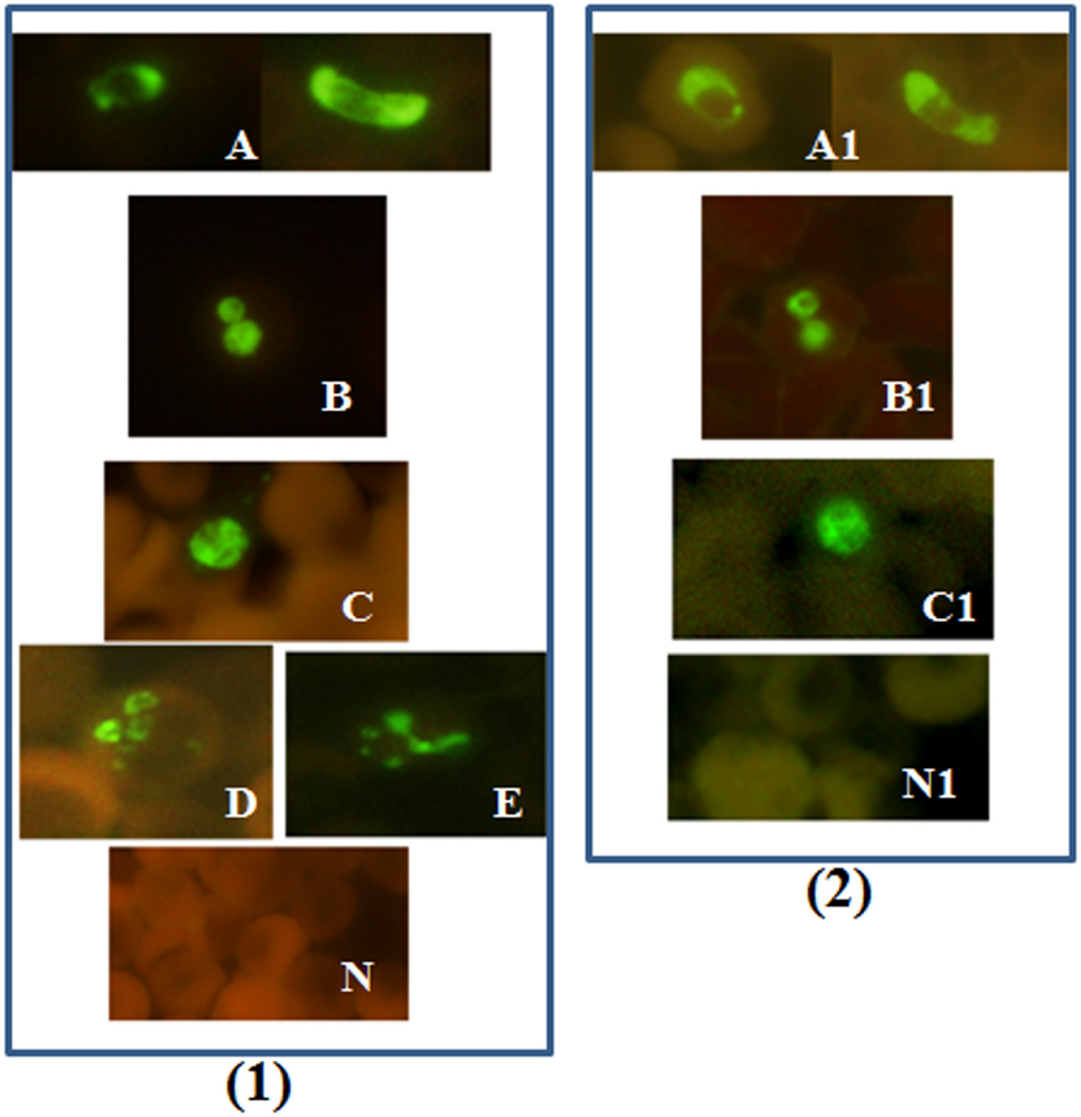
Blood smears tested with *Plasmodium* genus FISH assay. Blood smears from different *Plasmodium* species *P*. *falciparum*, *P*. *vivax*, *P*. *knowlesi*, *P*. *ovale and P*. *malariae* were analyzed using *Plasmodium* genus FISH assay. (1) All the processed smears were read with a 100X objective in a fluorescence microscope. (2) *P*. *falciparum*. *P*. *vivax*, *P*. *knowlesi* and negative control smears were read with a 100X objective on a regular microscope with a LED unit. Green fluorescence indicates the presence of *Plasmodium* ribosomal RNA (rRNA). (A) *P*. *falciparum* including crescent shaped gametocytes *(B) P*. *vivax*; (C) *P*. *knowlesi*; (D) *P*. *ovale*; (E) *P*. *malariae*; and (N) *Negative Control*; (A1) *P*. *falciparum*;. (B1) *P*. *vivax*; (C1) *P*. *knowlesi*; and (N1) *Negative Control*.

In the P-Genus FISH assay, the *Plasmodium* specific probe is labeled with an Alexa 488 green fluorescent dye. Therefore, all the *Plasmodium* parasites would appear green under the blue filter (Excitation 492 nm; emission 530 nm) when viewed in a fluorescence microscope (Fig 1). Normal controls and non-malaria parasites will not be visible under the blue filter (Fig 1).

**Fig 2:**
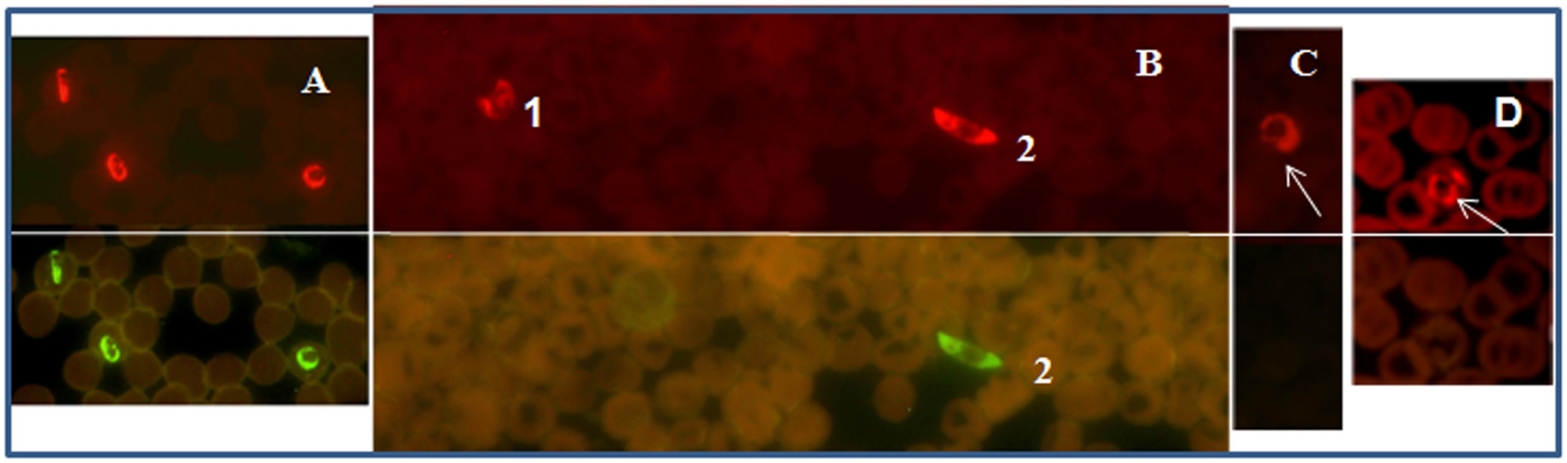
Blood smears tested with *P*. *falciparum* FISH assay. Malaria positive patient blood samples from two collection sites, Peru (A), and Kenya (B) were analyzed using PF-FISH assay. (A) Patient blood positive for *P*. *falciparum*. (B) Patient blood positive for *P*. *malariae* [1] *and P*. *falciparum* gametocyte [2]. (C) Patient blood positive for *P*. *ovale*. (D) Patient blood positive for *P*. *vivax*. Green fluorescence is due to the *P*. *falciparum* specific probe and red fluorescence due to the *Plasmodium* genus probe.

**Fig 3:**
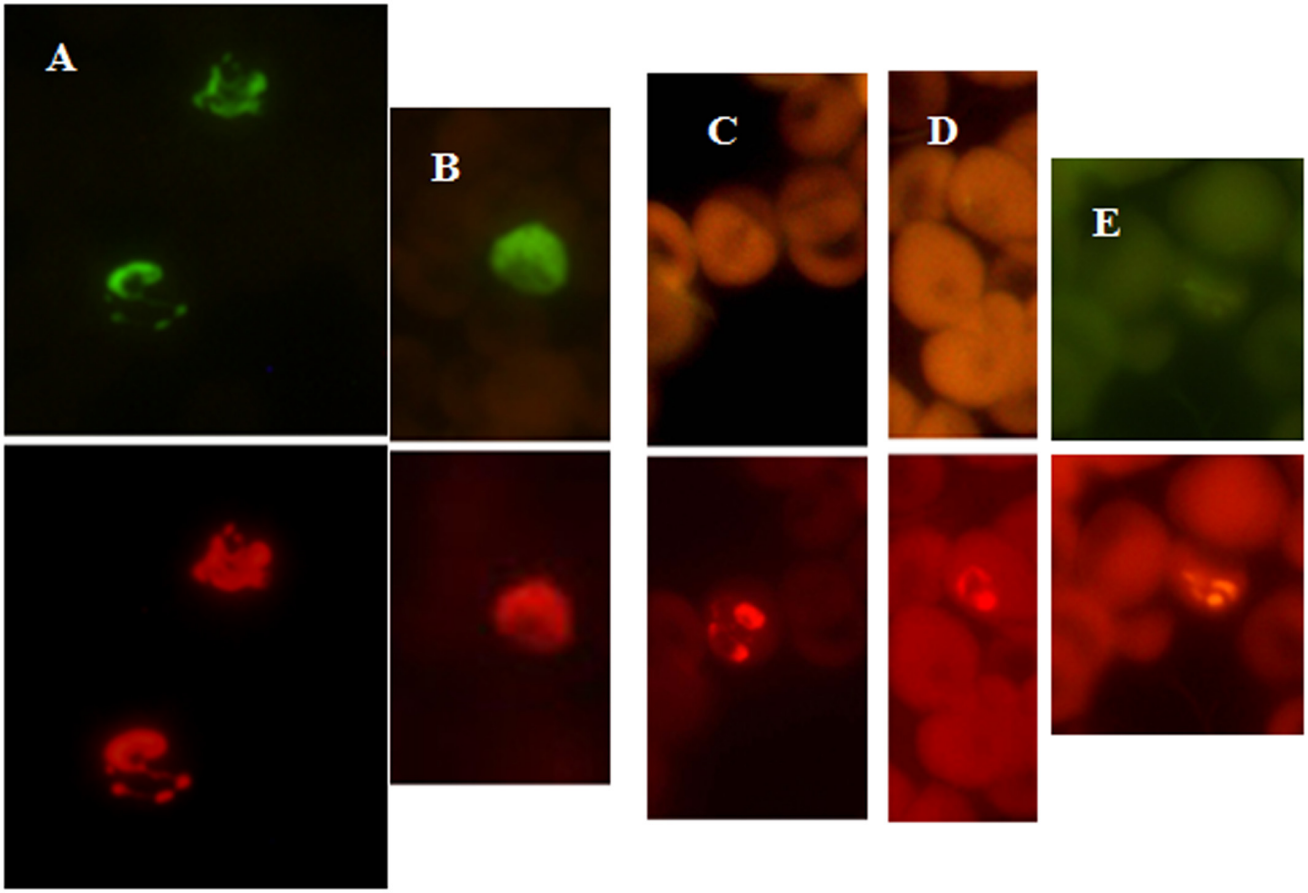
Blood smears tested with *P*. *vivax* FISH assay. Malaria positive patient blood samples from two collection sites, Peru (A), India (B) and Kenya (C-E) were analyzed using PV-FISH assay. (A) Patient blood positive for *P*. *vivax*. (B) Patient blood positive for *P*. *vivax*. (C) Patient blood positive for *P*. *ovale*. (D) Patient blood positive for *P*. *malariae*. (E) Patient blood positive for *P*. *falciparum*. Green fluorescence is due to reactivity with the *P*. *vivax* specific probe and red fluorescence is due to reactivity with the *Plasmodium* genus probe.

In the PF-FISH assay, the *P*. *falciparum* specific probe is labeled with the Alexa 488 dye and the *Plasmodium* genus probe is labeled with Texas Red dye; therefore only *P*. *falciparum* would appear green under the blue filter (Fig 2). Under the green filter (excitation 560; emission 630nm), all *Plasmodium* parasites, including *P*. *falciparum* will appear red (Fig 2). In the PV-FISH assay, *P*. *vivax* specific probe is labeled with the Alexa 488 dye and the *Plasmodium* genus probe is labeled with the Texas Red dye; therefore, only *P*. *vivax* would appear green under the blue filter (Fig 3). Under the green filter all the *Plasmodium* species will appear red (Fig 3). Normal controls and non-malaria parasites will not be visible under the blue filter or green filter with PF and PV FISH assay kits.

### Pathogenic organisms used in tests

*Borrelia burgdorferi* (ATCC B31), *Bartonella henselae* (ATCC 49882), *Leptospira interrogans* (ATCC 23476), *Plasmodium falciparum* (ATCC 30932), *P*. *vivax* (ATCC 30197) and *P*. *knowlesi* (ATCC 30158) cultures were purchased from ATCC (Atlanta, Georgia, USA). *Anaplasma phagocytophilum* and *Ehrlichia chaffeensis* cultures smears were provided by Prof. Stephen J. Dumler, John Hopkins University (Baltimore, Maryland). *Trypanosoma cruzi* culture smears and were provided by Dr. George L. Stewart, University of West Florida (Pensacola, Florida). Hamster blood infected with *Babesia duncani* and *Babesia microti* were purchased from Antibody Systems Inc, Texas. The study for infecting hamster blood with *B*. *microti* and *B*. *duncani* was approved by the Institutional Animal Care and Use Committee (IACUC) of University of West Florida, Pensacola, Florida. *Leishmania major* amastigotes and *Leishmania major* promastigotes smears, were provided by Kenya Medical Research Institute, Nairobi, Kenya. *P*. *malariae* positive and *P*. *ovale* positive human blood smears were provided by Walter Reed Project, Kisumu, Kenya.

### Evaluation of Light Emitting Diode (LED) light source with custom blue and green dual-filter for use in resource-poor settings

We also evaluated a LED light source (Fraen Corp, Cusago, Italy) with custom blue and green filter set that can be attached onto a regular light microscope for reading FISH smears. To evaluate the performance of the LED unit to read processed FISH smears, methanol-fixed smears were prepared from 19 patient blood samples and one *P*. *knowlesi* infected monkey blood sample. This included eight *P*. *falciparum* (4 high parasitaemia samples and 4 low parasitaemia samples); eight *P*. *vivax* (4 high parasitaemia and 4 low parasitaemia samples), one *P*. *knowlesi* and two controls. These smears were tested by P-Genus, PF and PV FISH assay kits and read on a standard mercury lamp fluorescence microscope with custom filters and a regular light microscope with a LED unit attached to it (Fig 1).

### Discrepant analysis by PCR

FISH results were compared with Giemsa results; and any samples that were positive by FISH but negative by Giemsa were further analyzed by PCR. PCR was performed on purified DNA with the following 2 primers Mal F2 5’- CGAAAGTTAAGGGAGTGAAGAC-3’ and Mal R2 5’-TCTCGCTTGCGCGAATACTCG-3’. The amplified DNA were tested by southern dot-blot with *P*. *falciparum* dig-probe, 5’-GTCACCTCGAAAGATGACTT-3’ and *P*. *vivax* dig probe, 5’-TAAACTCCGAAGAGAAAATTC-3’. The FISH positive sample was considered a “true positive” if it tested positive by PCR.

## Results

### Development of FISH assay for detection and differentiation of *P*. *falciparum* and *P*. *vivax* directly from blood smears

P-Genus FISH assay detected all the *Plasmodium* species tested, *P*. *falciparum*, *P*. *vivax*, *P*. *malariae*, *P*. *ovale* and *P*. *knowlesi* (Fig 1). PF-FISH and PV-FISH speciate the P-Genus positive samples to *P*. *falciparum* and *P*. *vivax* respectively (Figs 2 and 3). The FISH assays detect all stages of malaria parasites present in blood including gametocytes (Fig 1A).

### Analytical sensitivity of the FISH assays

The analytical sensitivity of the three FISH assays was determined with smears prepared from *P*. *falciparum* (ATCC 30932) and *P*. *vivax* (ATCC 30197) positive blood samples. Each parasite sample was diluted in a negative EDTA venous blood sample and tested using Giemsa stain to estimate the preliminary parasitemia. The estimated parasitemia by Giemsa in venous blood for the *P*. *falciparum* sample and for the *P*. *vivax* sample was 33,505 parasites/μl, and 7,271 parasites/μl respectively. After the parasitemia was determined, serial dilutions of the parasite sample into five uninfected donor venous blood samples were prepared. Methanol fixed smears were prepared from each diluted blood sample and tested by the three FISH assays. Limit of detection was defined as the lowest dilution at which >90% of the 35 smears tested gave a positive result. The limit of detection for venous blood was: 170 *P*. *falciparum* and 153 *P*. *vivax* parasites/μl of blood respectively, based on Giemsa results (Table 1). The limits of detection (parasites/μl blood) for FISH assays are summarized in Table 1. Serial dilution studies demonstrated that the number of parasites detected by FISH assay correlated linearly with parasitemia (*R* ^2^ of >0.997). Therefore, FISH assays could be expanded to estimate parasites/μl of blood in clinical samples as a semi-quantitative assay when positive controls are included as calibration standards.

**Table 1:** Lowest number of parasites detected per μl blood by Giemsa and FISH. N/A—not applicable.

	*P*. *falciparum* parasites/μl blood (range)	*P*. *vivax* parasites/μl blood (range)
**Giemsa**	170	153
**P-Genus FISH**	62 (25–101)	56 (16–95)
**PF-FISH**	55 (13–91)	N/A
**PV- FISH**	N/A	59 (26–92)

**Fig 4:**
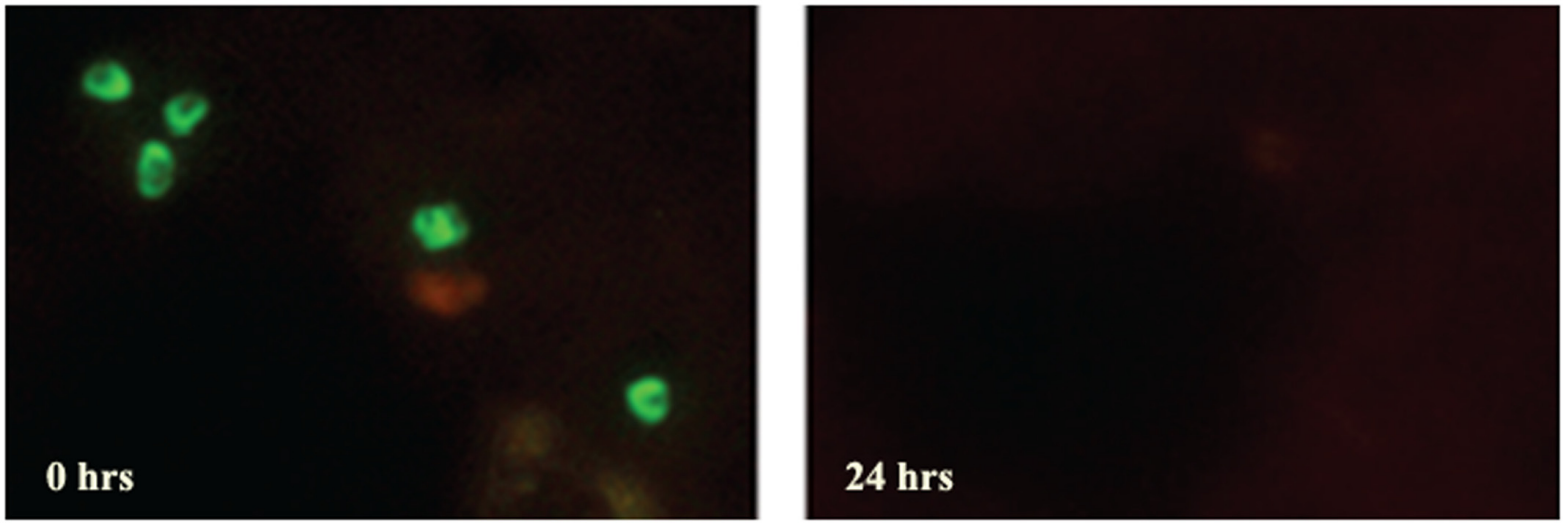
FISH assay only detects live *Plasmodium* parasites. Blood smears prepared from *P*. *falciparum* positive patient before (0 Hrs) and after 24 hours (24Hrs) drug treatment were analyzed by *Plasmodium* Genus FISH assay. The disappearance of the *Plasmodium* Genus fluorescence signal at 24 hours suggests that FISH assay only detects live *Plasmodium* parasites.

FISH detects live parasites, since rRNA, the target for FISH, has a short life and is only present in high copy numbers in a live organism [28]. In contrast, Giemsa and PCR detect both live and dead parasites. When blood smears from a patient positive for *P*. *falciparum* before, and 24 hours after treatment were tested with P-Genus FISH assay, the signal disappeared 24 hours after treatment (Fig 4), whereas Giemsa remained positive, further showing that FISH detects only live parasites.

### The FISH assays detect malaria parasites from different parts of the world

**Fig 5:**
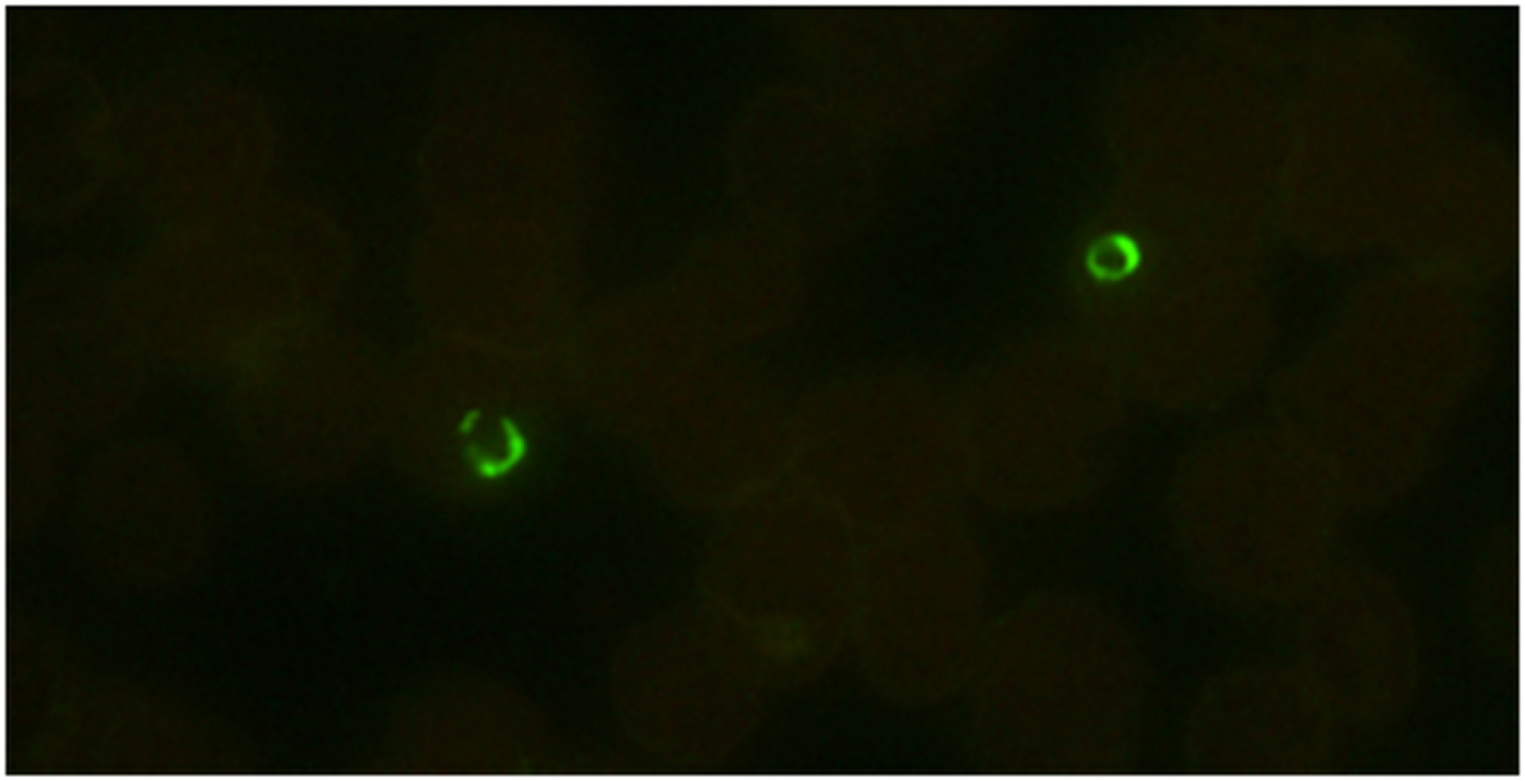
*P*. *falciparum* positive patient’s finger-prick capillary blood smear tested with *Plasmodium* genus FISH assay. Smear prepared from finger-prick capillary blood without anticoagulant. Green fluorescence indicates the presence of *Plasmodium* ribosomal RNA (rRNA) due to reaction with *Plasmodium* genus FISH.

Smears prepared from a set of 26 *Plasmodium* positive EDTA venous blood samples from different parts of the world ranging between 42–46,600 parasites per μl blood (Giemsa) were tested by P-Genus, PF and PV FISH assays. This included 25 human samples, 17 from Kenya [1 *P*. *falciparum*, 2 *P*. *ovale* and 14 *P*. *malariae*]; four from India [2 *P*. *falciparum* and 2 *P*. *vivax*]; four from Peru [2 *P*. *falciparum* and 2 *P*. *vivax*]) and one *P*. *knowlesi* (ATCC 30192), infected *Macaca fascicularis* blood sample. All 26 samples were detected by the P-Genus assay. PF-FISH assay only detected the 5 *P*. *falciparum* positive samples; and PV-FISH assay only detected the four *P*. *vivax* positive samples. (Details in S1 Table.) In addition, 14 patients’ left over methanol fixed thin smears, prepared from capillary blood drop from a finger prick without anticoagulant were tested by P-Genus FISH assay and read on light microscope with LED unit. This included 10 *P*. *vivax* positive samples and four *P*. *falciparum* positive samples by Giemsa. All the samples were positive by P-Genus FISH assay (Fig 5). This data clearly demonstrates that the *Plasmodium* FISH assays can detect *Plasmodium* parasites in patients’ blood smears and speciate *Plasmodium* positive samples to *P*. *falciparum* and *P*. *vivax*, irrespective of where the infection is acquired.

### Specificity of the three FISH assays for malaria parasites

To determine the analytical specificity of FISH assays, 22 pathogens’ smears were tested by all three FISH assays. This included 4 parasites (*Trypanosoma cruzi*, *Babesia duncani*, *B*. *microti and Leishmania major)*, *7 bacteria and 11 viruses* (listed in S2 Table). There was no cross-reaction to any of the pathogens tested.

### Reproducibility of the FISH assays

To test the reproducibility of the FISH assays, coded 54 panels, each consisting of six randomly selected patient samples, two *P*. *falciparum* positive samples, two *P*. *vivax* positive samples (1 high parasitaemia sample and 1 low parasitaemia sample of each species) and two negative samples for *Plasmodium* species were tested at three laboratories. Each panel was tested by two different operators at each site using three lots of the P-Genus, PF and PV FISH assays over a period of nine days.

For P-Genus and PV-FISH assays, the results were 100% accurate at all 3 sites. All the negative control samples tested negative by both assays. All the *Plasmodium* species positive samples tested positive by the P-Genus FISH assay and only *P*. *vivax* positive samples tested positive by PV-FISH kits. For the PF-FISH assay, all negative samples and high *P*. *falciparum* positives were tested accurately at all three sites. For the low *P*. *falciparum* positives, 4 of the 54 total smears tested negative, two each at two different sites. Overall 7.4% of the low *P*. *falciparum* samples tested negative. This corresponds to 92.6% agreement with the expected results near cut-off.

### Clinical Sensitivity and Specificity of the FISH assays

To further evaluate the FISH assays, we performed a clinical study using methanol-fixed smears prepared from 357 EDTA venous whole blood patient samples from patients ranging in age from 1 year to 65 years (302 were from KMC, Mangalore, India, 28 from Western Kenya and 27 from Iquitos, Peru), as well as 150 samples from individuals with malaria-like symptoms from the United States (USA), a region not endemic for malaria. The collecting sites also provided Giemsa and RDT results. Clinical study was performed at two sites. All the samples were tested by P-Genus FISH, PF-FISH and PV-FISH assay kits. Smears prepared from 302 blood samples collected at KMC were tested blinded at KMC; and the remaining patient smears were tested in our laboratory. FISH results were compared with Giemsa results and any samples that were positive by FISH but negative by Giemsa were further analyzed by PCR for discrepant analysis. The FISH positive sample was considered a “true positive” if it tested positive by PCR.

For the P-Genus FISH Assay, of the 357 samples tested, 152 were positive by Giemsa; 183 by FISH and 145 by RDT (Table 2). Using Giemsa as a reference method, the sensitivities of P-Genus FISH assay and RDT were 98% and 85.5% (Table 3); and the specificities were 83.4% and 92.7% respectively (Table 4). Seven out of 12 FISH and RDT positive samples and 10 out of 22 FISH-only positive samples were also positive for *Plasmodium* by PCR. These 17 samples were considered “true positives”. Thus after discrepant analysis, the sensitivities of P-Genus FISH, Giemsa and RDT were 98.2%, 89.9% and 81.1% (Table 3); and the specificities were 90.9%, 100% and 95.7%, respectively (Table 4).

**Table 2:** Summary of Clinical Study Results. Giemsa negative, PCR and FISH Positive samples a = 17; b = 5; c = 14. (Note: 150 samples from US are excluded)

	Giemsa	P-Genus FISH	RDT	Giemsa	PF FISH	RDT	Giemsa	PV FISH	RDT
**Positive**	152	183^a^	145	50	58^b^	57	102	130^c^	87
Negative	205	174	212	307	299	300	255	227	270

**Table 3:** Sensitivity of Plasmodium FISH tests and RDTs as compared to Giemsa. After discrepant analysis: Including FISH and PCR positive but Giemsa negative samples as true positives.” P-Genus FISH -17; PF-FISH– 5; and PV-FISH– 14. CI—confidence interval.

	Before Discrepant Analysis	After Discrepant Analysis
Test	Sensitivity (95% CI)	Sensitivity (95% CI)
P-Genus FISH	98.0 (93.9–99.5)	98.2 (94.5–99.5)
PF-FISH	94.0 (82.4–98.4)	94.5 (83.9–98.6)
PV-FISH	98.0 (92.4–99.7)	98.3 (93.3–99.7)
P-Genus RDT	85.5 (78.7–90.5)	81.1 (74.2–86.5)
PF RDT	94.4 (82.5–98.4)	94.5 (83.9–98.6)
PV RDT	79.4 (70.0–86.5)	73.3 (64.1–80.9)
P-Genus Giemsa	100	89.9 (84.1–93.9)
PF Giemsa	100	83.3 (71.0–91.3)
PV Giemsa	100	87.9 (80.2–93.0)

**Table 4:** Specificity of Plasmodium FISH tests and RDTs as compared to Giemsa. After discrepant analysis: Including FISH and PCR positive but Giemsa negative samples as true positives”. P-Genus FISH -17; PF-FISH– 5; and PV-FISH– 14. CI—confidence interval.

	Before Discrepant Analysis	After Discrepant Analysis
Test	Specificity (95% CI)	Specificity (95% CI)
P-Genus FISH	83.4 (77.4–88.0)	90.9 (85.7–94.5)
PF-FISH	96.4 (93.5–98.1)	98.0 (95.5–99.2)
PV-FISH	88.2 (83.5–91.8)	93.4 (89.2–96.0)
P-Genus RDT	92.7 (88.0–95.7)	95.7 (91.4–98.0)
PF RDT	96.7 (93.9–98.3)	98.3 (96.0–99.4)
PV RDT	97.6 (94.7–99.0)	99.2 (96.7–99.9)
P-Genus Giemsa	100	100 (97.5–100)
PF Giemsa	100	100 (98.0–100)
PV Giemsa	100	100 (98.4–100)

For the PF-FISH Assay, of the 357 samples tested, 50 were positive by Giemsa; 58 by FISH and 57 by RDT (Table 2). Using Giemsa as a reference method, the sensitivities of both PF-FISH assay and RDT were 94% (Table 3); and the specificities were 96.4% and 96.7% respectively (Table 4). Five out of nine FISH and RDT positive samples were also positive by *Plasmodium* PCR. These five samples were considered “true positives”. Thus after discrepant analysis, the sensitivities of PF-FISH, Giemsa and RDT were 94.5%, 83.3% and 94.5% (Table 3); and the specificities were 98%, 100% and 98.3%, respectively (Table 4).

For the PV-FISH Assay, of the 357 samples tested, 102 were positive by Giemsa; 130 by FISH and 87 by RDT (Table 2). Using Giemsa as a reference method, the sensitivities of PV-FISH assay and RDT were 98% and 79.4% (Table 3); and specificities were 88.2% and 97.6% respectively (Table 4). 14 samples, four out of five FISH and RDT positive samples and 10 out of 25 FISH only positive samples were also positive for *P*. *vivax* by PCR. These 14 samples were considered “true positives”. Thus after discrepant analysis, the sensitivities of PV-FISH, Giemsa and RDT were 98.3%, 87.9% and 73.3% (Table 3); and the specificities were 93.4%, 100% and 99.2% respectively (Table 4).

The specificity was 100% for all 3 FISH tests on 150 venous blood samples tested from US patients with malaria-like symptoms.

#### Evaluation of a LED light source with a custom blue and green filter set attached to a regular light microscope for FISH assays

As described in the methods section, smears prepared from 19 patients’ blood samples (8 *P*. *falciparum* positive; 8 *P*. *vivax* positive and 3 negatives) and one *P*. *knowlesi* infected monkey blood sample were tested by P-Genus, PF and PV-FISH assay kits and read on mercury fluorescence microscope and a regular light microscope with a LED unit attached to it. There was no difference in the reading between the two microscopes (Fig 1). All the 17 *Plasmodium* positive samples were positive by the P-Genus FISH. The eight *P*. *falciparum* positive samples were positive by PF-FISH and negative by PV-FISH; and the eight *P*. *vivax* positive samples were positive by the PV-FISH and negative by the PF-FISH. *P*. *knowlesi* positive blood sample was negative by both PF-FISH and PV-FISH.

## Discussion

In this study, we demonstrated that the *Plasmodium* FISH assays can be effective diagnostic tools for the detection of *Plasmodium* and speciation to *P*. *falciparum* and *P*. *vivax* in blood smears. The FISH assays are highly reproducible and specific. The limit of detection for the FISH assays, based on Giemsa results was between 153–174 parasites/μl blood and between 55–65 parasites/μl blood by FISH. This discrepancy can be attributed to the fact that Giemsa detects both live and dead parasites, whereas FISH detects only live parasites with intact rRNA [28]. FISH may be a superior tool for monitoring the efficacy of malaria treatment and avoiding unnecessary treatment, since it detects only live parasites; this in turn will avoid drug resistance. By enabling better clinical practice, the FISH assay could result in both short-term cost savings from un-indicated treatment and long-term improvements in the treatment of disease.

We have shown that P-Genus FISH assay detects the five species of parasites tested to-date, known to cause malaria in humans, namely, *P*. *falciparum*, *P*. *vivax*, *P*. *malariae*, *P*. *ovale* and *P*.*knowlesi* and the PF-FISH assay and PV-FISH assay detect and differentiate *P*. *falciparum* and *P*. *vivax* from other species of *Plasmodium*, respectively, on any P-Genus screen positive blood sample. Additionally, we observed that under Texas red filter dead parasites too were detected. Therefore the Texas Red fluorescent dye cannot be used for labeling species specific probes. Thus in the present study we performed PF-FISH and PV-FISH on separate smears.

We demonstrated that FISH assays are simple, and can detect all stages of the parasites. In addition, LED unit with blue-green filter attached to a regular light microscope with 100X objective can be used to read FISH processed smears instead of a mercury arc lamp fluorescence microscope. The assays consist of six steps: smear preparation, fixation, hybridization, washing, counterstaining and viewing the processed smear under a fluorescence microscope. The total assay time is approximately 60 minutes. The limit of detection is between 1–2 parasites per 300 fields with a 100X objective. This corresponds to 60–80 parasites/μl of blood. The FISH assays are reproducible and have the required sensitivity and specificity which is superior to that of the widely used microscopic examination of Giemsa stained blood smears.

FISH assays have other advantages over microscopic examination of Giemsa stained smears *viz*. (1) FISH detects specific 18S rRNA fragment in live parasites whereas by microscopy, Giemsa stained live and dead parasites are detected since parasite detection and speciation is based on the morphology only. (2) Since rRNA is present in the cytoplasm of the parasite, the whole cell fluoresces when viewed under the fluorescence microscope. Ribosomal rRNA is a unique molecule with highly conserved and variable regions. Thus, it is possible to design genus, species and strain specific probes. This unique property of rRNA, allows FISH assay to be equivalent in specificity to PCR. Although PCR is also very sensitive, it has limited utility in malaria endemic areas because of complex methodologies, high cost, and the need for trained technicians and special facilities. In addition equipment maintenance is also essential so that it may not be suitable for malaria diagnosis in remote rural areas or even in routine clinical diagnostic setting [5]. On the other hand FISH is relatively inexpensive as compared to PCR, since high maintenance equipment and temperature sensitive reagents are not required.

FISH assays using LED unit with appropriate filters attached to a regular light microscope instead of a mercury arc lamp fluorescence microscope may be the answer for diagnosis of malaria in resource-poor countries for the following reasons. (1) They are easy to perform and read, once the technique is mastered. (2) They are specific and have sensitivity equivalent to Giemsa smears read by experts. (3) They can differentiate species. (4) They provide morphological information. (5) They can be very useful for monitoring patients on treatment since they detect live parasites. (6) The LED unit with appropriate filters is relatively inexpensive and has following advantages. (i) It can be installed easily onto a regular microscope. (ii) It is very easy to use. (iii) It can operate on a rechargeable battery unit, making it possible to use in remote areas. (iv) There is no maintenance since LED has a lifetime of 10,000+ hours with no decaying curve. (v) Unlike mercury bulbs, LED light source does not require focus adjustments, nor post any health hazard.

One drawback of FISH is reading processed smears, since most technicians in malaria endemic regions currently are used to reading Giemsa smears and have no experience in fluorescent microscopy. Therefore, training programs similar to those for preparing and reading Giemsa smears would be required for the *Plasmodium* FISH assays. Despite the shortcoming noted, the FISH technique has a great potential for bridging the gap between high cost molecular tests and Giemsa microscopy for accurate diagnosis of malaria. This method can also be applied for detection of other pathogens present in blood. As such, this effort could represent a great potential contribution to the field of tropical medicine and infectious disease. The novelty of this method is not limited to the FISH assay alone, but also the innovative microscopic technology that is both cost-conscious and still technologically sound. Given the continued social and public health cost of malaria, these advances merit consideration for use by national and international health authorities.

## Ethics

### Human Subjects

The study was reviewed and approved by the Institutional Review Boards of Kasturba Medical College, Mangalore, India; Walter Reed-KEMRI, Kisumu, Kenya; Johns Hopkins Bloomberg School of Public Health, Bethesda, Maryland, USA; Universidad Peruana Cayetano Heredia, and Asociación Benéfica PRISMA, both in Lima, Peru; and by the Directorate of Health, Iquitos, Peru, Approval was granted to use archived de-identified samples that would otherwise be discarded. Patient consent was not required, since only left-over de-identified patient bloods samples submitted for routine testing, that would otherwise be discarded were used for the study.

### Animals

The study for infecting hamster blood with *B*. *microti* and *B*. *duncani* was approved by the Institutional Animal Care and Use Committee (IACUC) of University of West Florida, Pensacola, Florida.

## Supporting Information

S1 Table Inclusivity Study of *Plasmodium* Genus FISH assays. (DOC)

S2 Table Specificity Study. (DOC)

## References

[1] Ramasamy R. Zoonotic malaria—global overview and research and policy needs. Front Public Health. 2014;18 (2): 123–2:123. 10.3389/fpubh.2014.00123 PMC4135302 25184118

[2] WHO Geneva, Switzerland: WHO Press; 2. World Malaria Report 2014: 1–242,

[3] WHO Geneva, Switzerland: WHO Press; 2. World Malaria Report 2013: 1–255.

[4] CDC (2012) Malaria surveillance—United States, 2010. 1–17 p. 22377962

[5] Tangpukdee N, Duangdee C, Wilairatana P, Krudsood S. Malaria diagnosis: a brief review. Korean J Parasitol. 2009; 47: 93–102. 10.3347/kjp.2009.47.2.93 19488414 PMC2688806

[6] Joanny F, Löhr SJ, Engleitner T, Lell B, Mordmüller B. Limit of blank and limit of detection of Plasmodium falciparum thick blood smear microscopy in a routine setting in Central Africa. 2014 Malar J. 2014;13 (234) 10.1186/1475-2875-13-234 PMC4069274 24929248

[7] Meatherall B, Preston K, Pillai DR. False positive malaria rapid diagnostic test in returning traveler with typhoid fever. BMC Infect Dis. 2014; 14: 377 10.1186/1471-2334-14-377 25005493 PMC4094604

[8] Lee JH, Jang JW, Cho CH, Kim JY, Han ET, Yun SG, Lim CS. False-positive results for rapid diagnostic tests for malaria in patients with rheumatoid factor. J Clin Microbiol. 2014;52 (10):3784–7. 10.1128/JCM.01797-14 Epub 2014 Jul 23. 25056333 PMC4187788

[9] Baiden F, Webster J, Tivura M, Delimini R, Berko Y, Amenga-Etego S. et al Accuracy of rapid tests for malaria and treatment outcomes for malaria and non-malaria cases among under-five children in rural Ghana. PLoS ONE. 2012; 7(4): e34073 doi: 10.1371/ journal.pone.0034073 22514617 10.1371/journal.pone.0034073 PMC3325982

[10] Bjorkman A and Martensson A. Risks and benefits of targeted malaria treatment based on rapid diagnostic test results. CID 2010:51 Editorial Commentary 512–514. 10.1086/655689 20642355

[11] CDC Malaria rapid diagnostic test. 2007.

[12] Cullen KA and Arguin PM. Malaria Surveillance—United States. CDC Morbidity and Mortality Weekly Report Surveillance Summaries. 2012: 63 (12). 25474160

[13] Ndao M, Bandyayera E, Kokoskin E, Gyorkos TW, MacLean JD, Ward BJ. Comparison of blood smear, antigen detection, and nested-PCR methods for screening refugees from regions where malaria is endemic after a malaria outbreak in Quebec, Canada. J Clin Microbiol. 2004; 42:2694–700. 15184454 10.1128/JCM.42.6.2694-2700.2004 PMC427867

[14] Johnston SP, Pieniazek NJ, Xayavong MV, Slemenda SB, Wilkins PP, et al PCR as a confirmatory technique for laboratory diagnosis of malaria. Journal of Clinical Microbiol. 2006; 44: 1087–1089. 10.1128/JCM.44.3.1087-1089.2006 PMC1393165 16517900

[15] DeLong E. F., Wickham G. S., and Pace N. R.. 1989 Phylogenetic stains:ribosomal RNA—based probes for the identification of single cells. Science, 1989; 243:1360–1363. 2466341 10.1126/science.2466341

[16] Delong E, Shah JS (1990) Fluorescent, ribosomal RNA probes for clinical applications: A Research Review. Diagnosis and Clinical Testing 1990; 28(5): 41.

[17] Shah JS, Pieciak W, Liu J, Buharin A, Lane DJ. Diversity of host species and strains of Pneumocystis carinii is based on rRNA sequences. Clinical and Diagnostic Laboratory Immunology. 1996; 3: 119–127. 8770515 10.1128/cdli.3.1.119-127.1996 PMC170258

[18] Shah J. S. and Harris N. S. Detection of *Babesia microti* by Fluorescent In Situ-Hybridization. 39^th^ International Conference on Antimicrobial Agents and Chemotherapy, Sept 26–29 1999, San Francisco, CA.

[19] Stender H., Lund K., Petersen K.H., Rasmussen O.F., Hongmanee P., Miorner H., and Godtfredsen S.E. Fluorescence in situ hybridization assay using peptide nucleic acid probes for differentiation between tuberculosis and nontuberculosis mycobacterium species in smears of mycobacterium cultures. J.Clin. Microb.1999; 37:2760–2765. 10.1128/jcm.37.9.2760-2765.1999 PMC85371 10449448

[20] Stender H., Mollerup T.A., Lund K., Peterson K. H., Hongmanee P. and Godtfredsen S. E. 1999 Direct detection hybridization (FISH) using peptide nucleic acid (PNA) probes. Int. J. Tuberc. Lung Dis 3. 1999; 9:830–837. 10488893

[21] Amand AL St, Frank DN, De Groote MA, Basaraba RJ, Orme IM, Pace NR. Use of specific rRNA oligonucleotide probes for microscopic detection of Mycobacterium tuberculosis in culture and tissue specimens. J Clin Microbiol. 2005;43(10):5369–71. 16208021 10.1128/JCM.43.10.5369-5371.2005 PMC1248482

[22] Amand AL St, Frank DN, De Groote MA, Pace NR. Use of specific rRNA oligonucleotide probes for microscopic detection of Mycobacterium avium complex organisms in tissue. J Clin Microbiol. 2005; 43(4):1505–14. 15814959 10.1128/JCM.43.4.1505-1514.2005 PMC1081365

[23] Lefmann M, Schweickert B, Buchholz P, Göbel UB, Ulrichs T, Seiler P, Theegarten D, Moter A. Evaluation of peptide nucleic acid-fluorescence in situ hybridization for identification of clinically relevant mycobacteria in clinical specimens and tissue sections. J Clin Microbiol. 2006; 44 (10):3760–7. 17021106 10.1128/JCM.01435-06 PMC1594750

[24] Rigby S, Procop GW, Haase G, Wilson D, Hall G, Kurtzman C, Oliveira K, Von Oy S, Hyldig-Nielsen JJ, Coull J, Stender H. Fluorescence in situ hybridization with peptide nucleic acid probes for rapid identification of Candida albicans directly from blood culture bottles. J Clin Microbiol. 2000; 40(6):2182–6. 10.1128/JCM.40.6.2182-2186.2002 PMC130801 12037084

[25] Machado A, Almeida C, Salgueiro D, Henriques A, Vaneechoutte M, Haesebrouck F, Vieira MJ, Rodrigues L, Azevedo NF, Cerca N.2013 Fluorescence in situ Hybridization method using Peptide Nucleic Acid probes for rapid detection of Lactobacillus and Gardnerella spp. 2013; BMC Microbiology 13:82 10.1186/1471-2180-13-82 23586331 PMC3637831

[26] Shah JS, Harris NS. In Situ- Hybridization for detecting target nucleic acid. US Patent No. 6,165,723. 2000.

[27] Woese CR. Bacterial Evolution. Microbiological Reviews 1987; 51: 221–271. 2439888 10.1128/mr.51.2.221-271.1987 PMC373105

[28] Graczyk TK, Grimes BH, Knight R, Da Silva AJ, Pieniazek NJ, et al Detection of Cryptosporidium parvum and Giardia lamblia carried by synanthropic flies by combined fluorescent in situ hybridization and a monoclonal antibody. Am. J. Trop. Med. Hyg. 2003; 68: 228–232. 12641416

