# Supplementary material for: Fluorescence In Situ Hybridization (FISH) Assays for Diagnosing Malaria in Endemic Areas
Source: PLoS One. 2015 Sep 2;10(9):e0136726. doi: 10.1371/journal.pone.0136726 (PMC4558036; doi:10.1371/journal.pone.0136726)
Supplement: S1 Table — (DOC) [file pone.0136726.s001.doc]

| **S1 Table: Inclusivity Study of *Plasmodium* Genus FISH assays** | | | | | | |
| --- | --- | --- | --- | --- | --- | --- |
| **Sample Type** | **Source** | **Organism** | **No. of parasites/µl blood (Giemsa)** | **FISH Results** | | |
|  |  |  |  | **P-Genus** | **PF** | **PV** |
| **Clinical Samples** | India | *P. falciparum* | 249 | Pos | Pos | Neg |
|  | India | *P. falciparum* | 5600 | Pos | Pos | Neg |
|  | India | *P. vivax* | 640 | Pos | Neg | Pos |
|  | India | *P. vivax* | 3160 | Pos | Neg | Pos |
|  | Peru | *P. falciparum* | 4457 | Pos | Pos | Neg |
|  | Peru | *P. falciparum* | 13521 | Pos | Pos | Neg |
|  | Peru | *P. vivax* | 5370 | Pos | Neg | Pos |
|  | Peru | *P. vivax* | 2670 | Pos | Neg | Pos |
|  | Kenya | *P. falciparum* | 61 | Pos | Pos | Neg |
|  | Kenya | *P. ovale* | 2472 | Pos | Neg | Neg |
|  | Kenya | *P. ovale* | 51 | Pos | Neg | Neg |
|  | Kenya | *P. malariae* | 1346 | Pos | Neg | Neg |
|  | Kenya | *P. malariae* | 315 | Pos | Neg | Neg |
|  | Kenya | *P. malariae* | 20160 | Pos | Neg | Neg |
|  | Kenya | *P. malariae* | 441 | Pos | Neg | Neg |
|  | Kenya | *P. malariae* | 63 | Pos | Neg | Neg |
|  | Kenya | *P. malariae* | 63 | Pos | Neg | Neg |
|  | Kenya | *P. malariae* | 945 | Pos | Neg | Neg |
|  | Kenya | *P. malariae* | 294 | Pos | Neg | Neg |
|  | Kenya | *P. malariae* | 651 | Pos | Neg | Neg |
|  | Kenya | *P. malariae* | 630 | Pos | Neg | Neg |
|  | Kenya | *P. malariae* | 126 | Pos | Neg | Neg |
|  | Kenya | *P. malariae* | 609 | Pos | Neg | Neg |
|  | Kenya | *P. malariae* | 14049 | Pos | Neg | Neg |
|  | Kenya | *P. malariae* | 42 | Pos | Neg | Neg |
| Monkey blood | ATCC | *P. knowlesi* | 46600 | Pos | Neg | Neg |
| Methanol fixed blood smears prepared from EDTA whole blood, were tested with P-Genus, PF and PV FISH assay kits. | | | | | | |
