# Supplementary material for: Fluorescence In Situ Hybridization (FISH) Assays for Diagnosing Malaria in Endemic Areas
Source: PLoS One. 2015 Sep 2;10(9):e0136726. doi: 10.1371/journal.pone.0136726 (PMC4558036; doi:10.1371/journal.pone.0136726)
Supplement: S2 Table — (DOC) [file pone.0136726.s002.doc]

| **S2 Table: Specificity Study *Plasmodium* FISH assays** | | | | | | |
| --- | --- | --- | --- | --- | --- | --- |
| **TYPE** | **PATHOGEN TESTED** | **CONCENTRATION TESTED** | **P-Genus FISH** | **PF-FISH** | **PV-FISH** |  |
| **Parasites** | *Trypanosoma cruzi* culture (UCF) | 1 x 10^7^ parasites/ml | Negative | Negative | Negative |  |
|  | *Babesia duncani* (AS Inc) | 3.5 x 10^6^ parasites/ml | Negative | Negative | Negative |  |
|  | *Babesia microti* (AS Inc) | 3.5 x 10^6^ parasites/ml | Negative | Negative | Negative |  |
|  | *Leishmania major* - amastigotes (KEMRI- NBO) | 1.5 x 10^6^ parasites/ml | Negative | Negative | Negative |  |
|  | *Leishmania* *major* - promastigotes (KEMRI- NBO) | > 1 x 10^7^ parasites/ml | Negative | Negative | Negative |  |
| **Viruses** | *Epstein-Barr virus* (KEMRI-NBO) | > 5 x 10^5^ copies/ml | Negative | Negative | Negative |  |
|  | *Yellow fever virus* (KEMRI-NBO) | > 1 x 10^7^ PFU/ml | Negative | Negative | Negative |  |
|  | *Dengue virus* (KEMRI-NBO) | > 1 x 10^7^ PFU/ml | Negative | Negative | Negative |  |
|  | *Chikungunya virus* (KEMRI-NBO) | 4.1 X 10^7^ PFU/ml. | Negative | Negative | Negative |  |
|  | *Bwamba virus* (KEMRI-NBO) | > 1 x 10^7^ PFU/ml | Negative | Negative | Negative |  |
|  | *West Nile virus* (KEMRI-NBO) | > 1 x 10^7^ PFU/ml | Negative | Negative | Negative |  |
|  | *Cytomegalovirus* (UCSF) | > 5 x 10^5^ copies/ml | Negative | Negative | Negative |  |
|  | *Influenza virus A* (UCSF) | Unknown titer | Negative | Negative | Negative |  |
|  | *Influenza virus B* (UCSF) | Unknown titer | Negative | Negative | Negative |  |
|  | HIV-1 - patient blood (UCSF) | 7.6 X 10^4^ copies/ml | Negative | Negative | Negative |  |
|  | *Hepatitis B* -patient blood (KMC, India) | Infected patient blood | Negative | Negative | Negative |  |
| **Bacteria** | *Borrelia burgdorferi* - ATCC B31 strain (IGeneX) | 1 x 10^7^ bacteria/ml | Negative | Negative | Negative |  |
|  | *Bartonella henselae* (ATCC 49882)* | >3 x 10^7^ bacteria/ml | Negative | Negative | Negative |  |
|  | *Rickettsia rickettsii* (Focus) | >1x 10^7^ bacteria/ml | Negative | Negative | Negative |  |
|  | *Rickettsia typhi* (Focus) | >1x10^7^ bacteria/ml | Negative | Negative | Negative |  |
|  | *Anaplasma phagocytophilum* (HOPKINS) | 2.8x10^5^ bacteria/ml | Negative | Negative | Negative |  |
|  | *Ehrlichia chaffeensis* (HOPKINS) | 7.3x10^4^ bacteria/ml | Negative | Negative | Negative |  |
|  | Leptospira interrogans (ATCC 23476) | >1 x 10^6^ bacteria/ml | Negative | Negative | Negative |  |
| **Positive Controls** | *Plasmodium falciparum* infected whole blood | | Positive | Positive | Negative |  |
|  | *Plasmodium vivax* infected whole blood | | Positive | Negative | Positive |  |
| **Negative Control** | Normal human whole blood | | Negative | Negative | Negative |  |
